# Spinal Circuits Transmitting Mechanical Pain and Itch

**DOI:** 10.1007/s12264-017-0136-z

**Published:** 2017-05-08

**Authors:** Bo Duan, Longzhen Cheng, Qiufu Ma

**Affiliations:** 1 Department of Molecular, Cellular, and Developmental Biology, University of Michigan, 830 North University Ave, Ann Arbor, MI 48109 USA; 2 Institute of Brain Science, the State Key Laboratory of Medical Neurobiology and the Collaborative Innovation Center for Brain Science, Fudan University, Shanghai, 200032 China; 3 Dana-Farber Cancer Institute and Department of Neurobiology, Harvard Medical School, 1 Jimmy Fund Way, Boston, MA 02115 USA

**Keywords:** Pain, Itch, Gate control, Spinal cord

## Abstract

In 1905, Henry Head first suggested that transmission of pain-related protopathic information can be negatively modulated by inputs from afferents sensing innocuous touch and temperature. In 1965, Melzak and Wall proposed a more concrete gate control theory of pain that highlights the interaction between unmyelinated C fibers and myelinated A fibers in pain transmission. Here we review the current understanding of the spinal microcircuits transmitting and gating mechanical pain or itch. We also discuss how disruption of the gate control could cause pain or itch evoked by innocuous mechanical stimuli, a hallmark symptom for many chronic pain or itch patients.

Management of chronic pain and itch remains a major medical challenge. One common symptom seen in these patients is the presence of allodynia or alloknesis-pain or itch evoked by innocuous mechanical stimuli [1–10]. Chronic pain can be caused by tissue inflammation (inflammatory pain) or by lesions of the nervous system (neuropathic pain). Studies in the past decades have revealed many mechanisms leading to allodynia. In one scenario, peripheral sensitization following inflammation allows high-threshold nociceptors to gain the ability to respond to innocuous mechanical stimuli (for details, see the recent reviews [11–15]). The other scenario is partly based on the gate control theory first postulated by Ronald Melzak and Patrick Wall in 1965 and then revised in subsequent years [5, 16, 17], allowing low-threshold mechanoreceptors (LTMRs) to activate pain transmission neurons under pathological conditions. In this mini-review, we provide an update on the identities of spinal neurons that form the microcircuits underlying the gate control of mechanical pain or itch.

## Pain Theories and Mapping Dorsal Spinal Circuits

**Fig. 1:**
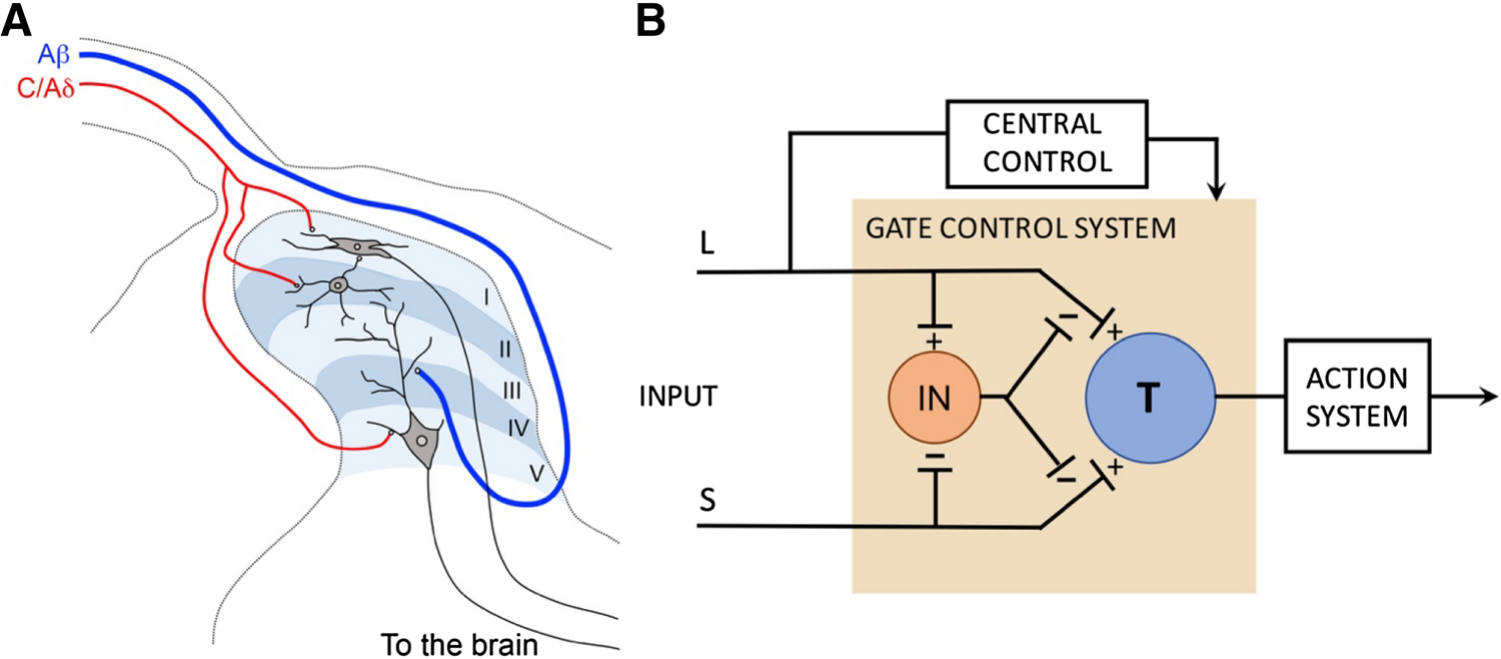
Laminar organization of the spinal dorsal horn. **A** Laminar organization of dorsal horn and primary afferent inputs, modified from Craig [19]. **B** The gate control theory proposed in 1965. T, transmission neurons; IN, inhibitory neurons in the substantia gelatinosa (lamina II) of the dorsal horn.

The mammalian dorsal spinal cord transmits and processes information related to a variety of sensory modalities, including pain, itch, temperature, and touch [5, 18]. It is organized into distinct laminae [18, 19]. Unmyelinated C afferents and thinly-myelinated Aδ sensory afferents that transmit pain, itch, and temperature primarily terminate in laminae I/II, as well as lamina V and other more ventral laminae [5, 18]. Various classes of LTMRs, including myelinated Aβ fibers, as well as Aδ and C fibers, terminate from the ventral inner layer of lamina II (vIIi) to lamina V [20]. The major output neurons include projection neurons located in lamina I and laminae III-VI, which ascend along the anterolateral tract or through the dorsal column [18, 21] (Fig. 1A).

One key question in the somatosensory field is to understand how the dorsal horn transmits distinct sensory modalities. Four different theories, including the specificity *versus* gate control theories discussed here, have been proposed in past centuries, as recently reviewed by Perl and others [22–25]. The specificity hypothesis suggests the existence of specific neural circuits transmitting different sensory modalities. Regarding pain-related information transmission, the specificity hypothesis is supported by the discovery of nociceptive-specific (NS) neurons in the late 1960s and early 1970s, based on extracellular recordings [26] (but see also below). Primary afferents expressing the G-protein coupled receptor (GPCR) MrgrpA3 and spinal neurons expressing the gastrin-releasing peptide receptor (GRPR) are required selectively to transmit chemical itch, but not pain, providing further support for the specificity hypothesis [27–30]. The gate control theory discussed in this review highlights crosstalk among different afferents in shaping sensory information transmission. Sensory afferent crosstalk is clearly suggested by the Thermal-Grill illusion discovered in 1896, showing that cold and warm stimulations in alternative skin regions generate paradoxical hot or even burning pain percepts [31, 32]. In 1905, Henry Head performed nerve lesion on his own hand, and, based on the differential regeneration speeds of different sensory afferents and the progressive change in perception in response to various sensory stimuli, he concluded that the crude protopathic pain perception generated by noxious stimuli can be attenuated by inputs from epicritic afferents that sense innocuous touch and temperatures [33], leading to the prototype of the gate control theory. In 1965, Melzack and Wall then proposed a more concrete gate control theory that was built on both clinical observations and electrophysiological recordings [16]. Gate control theory has several features (Fig. 1B), following incorporation of the discovery of the large number of nociceptors by Perl and his colleagues. First, spinal transmission (“T”) neurons normally receive inputs from nociceptors, and their activation evokes pain and other action systems. Second, transmission of nociceptive information to T neurons can be modulated by descending inputs from various brain regions. Third, T neurons concurrently receive excitatory inputs from LTMRs, but these inputs are gated *via* feedforward activation of local inhibitory interneurons (“IN”), such that innocuous mechanical stimuli normally suppress acute nociceptive pain. Fourth, strong nociceptive inputs, as well as plasticity induced by inflammation or nerve injury, somehow attenuate the inhibitory inputs from IN neurons and/or sensitize T neurons, such that normally subthreshold LTMR inputs can now sufficiently activate T neurons to evoke allodynia (Fig. 1B).

Recent years have seen important progress in characterizing the spinal circuits that transmit mechanical pain or itch. Together with the Martyn Goulding lab at the Salk Institute, we have been using an intersectional genetic strategy to identify spinal neurons involved in the transmission and gating of distinct modalities [34–36]. This intersectional genetic strategy allows us to ablate or silence specific spinal neurons that are defined by co-expression of the Cre DNA recombinase driven from a specific gene and the Flpo recombinase driven from the *Lbx1* gene whose expression is restricted to the dorsal spinal cord and dorsal hindbrain [37, 38]. As such, only dorsal spinal/hindbrain excitatory or inhibitory neurons that co-express X^Cre^ (X indicates a specific gene) and Lbx1^Flpo^ are ablated or silenced, without affecting Cre-expressing neurons in the peripheral nervous system or in the brain [34–36]. Meanwhile, several labs combined genetic and viral tools to manipulate specific populations of spinal neurons [39–42]. Subsequent behavioral and electrophysiological studies have now provided considerable insights into the transmission of mechanical pain and/or itch in the dorsal spinal cord. In this review, we focus on the identities of T neurons for the transmission of mechanical pain, the pathways linking LTMRs to T neurons, the characterization of IN neurons for the gate control of mechanical pain or itch, and the identities of relevant LTMRs.

## Spinal “T” Neurons Transmitting Mechanical Pain

**Fig. 2:**
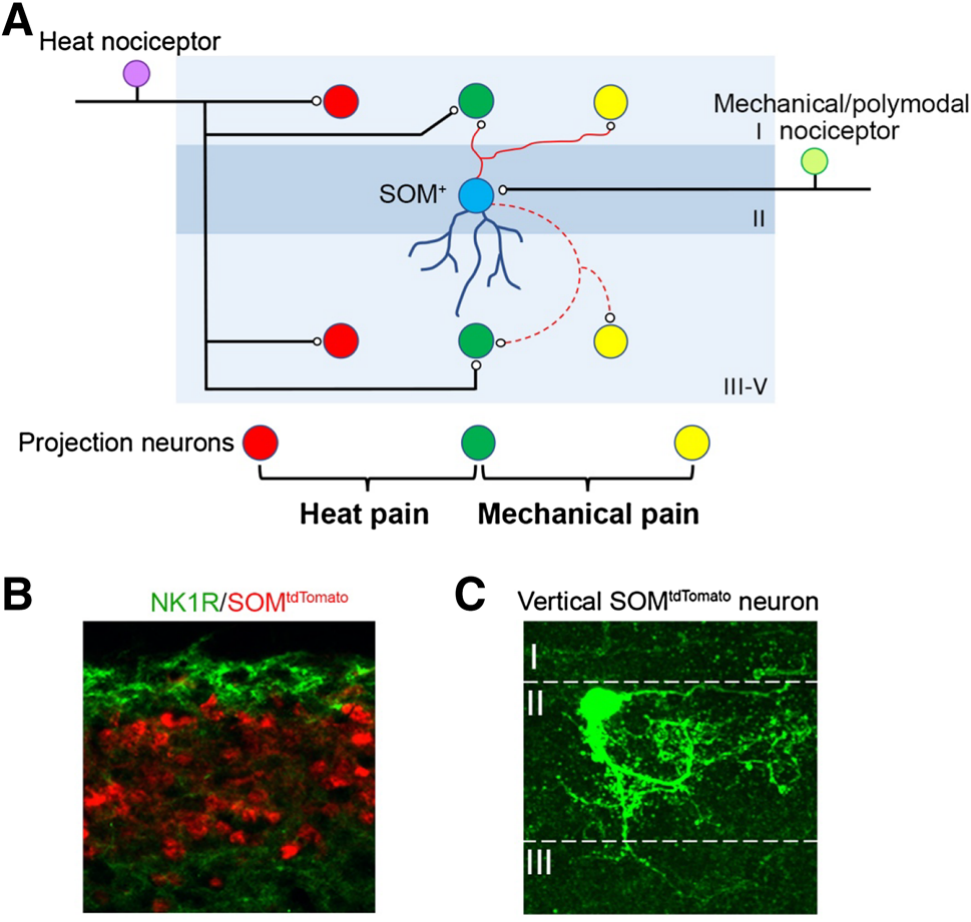
Transmission of mechanical *versus* heat pain. **A** Three types of projection neuron located in lamina I and deep laminae, including mechanical-selective (*yellow*), heat-selective (*red*), and polymodal (*green*) projection neurons. **B**, **C** SOM lineage neurons (marked after crossing SOM^Cre^ with tdTomato reporter mice, as previously described [34]), are enriched in laminae II and III, ventral to NK1R^+^ projection neurons in lamina I (**B**). Biocytin labeling showing a SOM^tdTomato^ neuron in lamina II that is a vertical cell (**C**). Image in C adapted from Duan *et al*. [34].

Spinal “T” neurons for mechanical pain transmission are defined as excitatory neurons that receive monosynaptic inputs from mechano-sensitive nociceptors. “T” neurons do not necessarily represent ascending projection neurons as suggested by the original gate control diagram [16]. We found that spinal neurons in the somatostatin (SOM) lineage, marked by SOM^Cre^ in which the Cre recombinase is driven from the *SOM* gene locus, are enriched in laminae II and III and critical for the transmission of both acute and chronic mechanical pain. Mice with intersectional ablation of a major subset (~85%) of SOM lineage neurons defined by the co-expression of SOM^Cre^ and Lbx1^Flpo^, referred to as SOM^Lbx1^ neurons, fail to respond to noxious mechanical stimuli. Surprisingly, SOM^Lbx1^ neurons are dispensable for nocifensive behaviors evoked by noxious thermal stimuli, even though many primary afferents and spinal output neurons in superficial dorsal horn laminae are polymodal, responding to both noxious heat and mechanical stimuli [34]. In order to explain the nearly complete loss of cutaneous mechanical pain in SOM^Lbx1^ neuron-ablated mice, inputs from both mechanical-selective nociceptors and mechanically sensitive polymodal nociceptors should all be transmitted *via* SOM^Lbx1^ neurons in lamina II (Fig. 2), whereas inputs from heat-selective sensory fibers, marked by the transient receptor potential channel TRPV1 (ref. [43, 44]), must be sufficient to mediate heat-evoked nocifensive responses *via* direct projection to heat-selective or polymodal neurons in laminae I/II_o_ or deep laminae that do not belong to the SOM lineage [26, 45] (Fig. 2). Earlier studies suggested that vertical cells represent major output neurons that relay sensory information from lamina II to lamina I [46–51], and consistently, the SOM lineage neurons include vertical cells with elaborate dendritic trees reaching laminae II–IV and a thin axon projecting to lamina I. SOM^Lbx1^ neurons could, however, play a redundant role in transmitting heat pain-related information, by relaying inputs from polymodal nociceptors. Consistent with these findings, Christensen* et al*. activated spinal SOM^+^ interneurons in adults using optogenetics, resulting in a nocifensive licking response [52]. Chemogenetic inhibition of SOM^+^ neurons reduced acute mechanical sensitivity and mechanical allodynia following peripheral inflammation [52]. However, they also found a slight deficit in noxious thermal sensation by chemogenetic inhibition of spinal SOM^+^ neurons [52], suggesting the possibility that a subset of SOM^Cre^ neurons that do not express Lbx1^Flpo^, which would be preserved in SOM^Lbx1^ neuron-ablated mice, might play a role in mediating thermal pain.

Primary afferents transmitting noxious mechanical information have recently been characterized. Unmyelinated primary sensory afferents expressing the G-protein-coupled receptor MrgprD are required to respond to light punctate mechanical stimuli evoked by von Frey filaments [53], whereas myelinated afferents expressing the neuropeptide Y receptor NPY2R transmit superthreshold pinprick-evoked intense mechanical pain [54]. In the dorsal spinal cord, with the existence of wide dynamic range (WDR) neurons, it had long been postulated that mechanical pain intensity might be encoded by the firing rates of WDR neurons [55, 56]. However, characterization of other molecularly defined spinal neurons shows that light punctate mechanical information, but not pinprick-evoked intense mechanical information, is transmitted through spinal neurons marked by the developmental expression of calbindin 2 (Calb2, also known as calretinin), which is in contrast to the transmission of both light and intense mechanical pain by the SOM lineage neurons. Lineage tracing experiments have shown that the Calb2 lineage neurons partially belong to the SOM lineage, albeit to those with transient SOM expression [34]. Conceivably, MrgprD^+^ neurons and NPY2R^+^ Aδ mechanical nociceptors might be preferentially connected to the Calb2 lineage neurons and the Calb2-negative SOM^Lbx1^ neurons to transmit light and intense mechanical pain, respectively. This initial segregation, however, does not necessarily argue against the intensity-encoding hypothesis. For example, Calb2^+^ neurons and Calb2-negative SOM^Lbx1^ neurons could send convergent inputs to downstream WDR neurons.

## Multiple Gated Spinal Pathways Linking LTMR Inputs to Lamina I Output Neurons

**Fig. 3:**
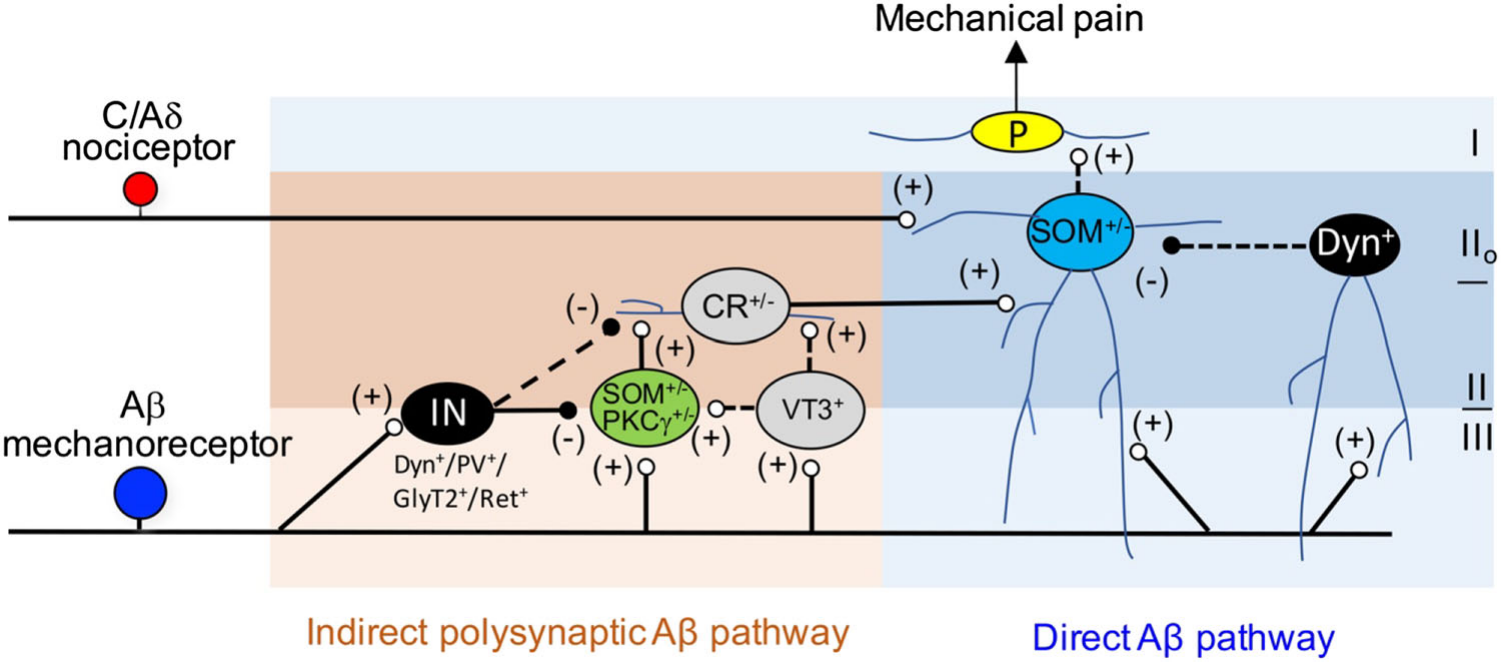
Schematic showing the spinal circuits that transmit mechanical pain-related information. CR^+/−^, transient-central cells partly marked by Calb2/calretinin^Cre^ (CR^+/−^); SOM^+/−^ cell (*blue*), vertical neuron in lamina II_o_; P, projection neuron in lamina I; IN, inhibitory interneuron at the II-III border or within lamina III, including the Dyn, PV, GlyT2, and Ret lineage neurons. Modified from Duan *et al*. [34].

A key prediction from the gate control theory is that spinal T neurons receive inputs not only from nociceptors, but also from Aβ-LTMRs (and possibly Aδ-LTMRs as well), and LTMR inputs are gated *via* feedforward activation of inhibitory neurons under normal conditions. Light and Perl initially performed extracellular recordings in laminae I/II_o_, revealing many nociception-specific neurons [47]. However, extracellular recordings can only detect inputs sufficient to generate an action potential output. Subsequent *in vivo* intracellular recordings then revealed subthreshold inputs from innocuous mechanical stimuli [57, 58], and multiple pathways relay LTMR inputs from lamina III to laminae I/II_o_ (Fig. 3). First, vertical cells in laminae I/II_o_, including SOM neurons that represent T neurons for acute mechanical pain transmission, send dendrites to laminae III/IV and receive direct (not necessarily monosynaptic) inputs from A-LTMRs. Second, electrophysiological recordings also reveal polysynaptic pathways starting with neurons located at the II–III border or within lamina III [34, 41, 59–61]. SOM neurons at the II–III border partially overlap with neurons expressing protein kinase C gamma (PKCγ), which receive detectable monosynaptic or polysynaptic Aβ inputs with or without action potential firing due to feedforward inhibition [34, 40, 61–63]. Peirs* et al*. subsequently reported that lamina III neurons marked by transgenic *Vglut3::Cre*, most of which receive monosynaptic inputs from Aβ fibers, play a critical role in relaying Aβ inputs to superficial laminae as well [41]. Since neurons at the II–III border or within lamina III normally do not receive nociceptive afferent inputs, at least based on extracellular recordings [47], the neurons that receive gated Aβ inputs no longer fit the original “T” neurons defined to be involved in the transmission of acute mechanical pain. Because the relay of Aβ fiber inputs from lamina III to lamina I is virtually abolished in mice with ablation of SOM lineage neurons, Vglut3::Cre-marked neurons could either overlap with SOM neurons or are connected to SOM neurons located in laminae II to relay Aβ inputs. Indeed, chemical genetic activation of Vglut3::Cre-marked neurons is able to activate PKCγ neurons and Calb2^+^ neurons [41], both of which partially belong to the SOM lineage [34] (Fig. 3).

The direct and polysynaptic Aβ pathways are gated *via* feedforward activation of inhibitory neurons located in laminae I–III. The dorsal pathway is gated at least partly *via* spinal inhibitory neurons marked by the Cre driven from the preprodynorphinin locus (Dyn^Cre^). The Dyn^Cre^ labels mainly inhibitory neurons enriched in laminae I and II, a subset of which represents vertical cells that receive Aβ inputs with action potential firing [34]. Following ablation of the Dyn lineage inhibitory neurons, most superficial dorsal horn neurons in I/II_o_ now receive Aβ inputs with action potential firing, a third of which are monosynaptic, indicating the opening of the direct pathway as well as polysynaptic pathways. A number of inhibitory neurons have been shown to gate the polysynaptic Aβ pathways, and these inhibitory neurons are enriched in laminae II and III, including inhibitory neurons marked by Cre driven from the parvalbumin gene locus (PV^Cre^) and the receptor tyrosine kinase Ret gene locus (Ret^CreER^), as well as the glycine transporter gene locus (GlyT2^Cre^) [39, 40, 42] (Fig. 3). Dyn neurons also contribute to the gating of the polysynaptic Aβ pathways [34] (Fig. 3). Strikingly, individual ablation of these four sets of genetically marked inhibitory neurons leads to the spontaneous manifestation of mechanical allodynia. We envision several scenarios: (1) these four sets of cells could overlap with each other and the overlapping portion gates the same allodynia pathways, (2) a summation of inputs from all these inhibitory neurons is needed to gate a pathway, and (3) perhaps most likely, these inhibitory neurons may gate distinct allodynia pathways, opening each of which would be sufficient to allow low-threshold mechanical stimuli to evoke pain.

Peripheral inflammation and nerve injury cause central sensitization and disinhibition, which collectively lead to gate opening and the manifestation of allodynia [5, 6, 12, 13, 25, 64–66]. Disinhibition can occur *via* many different mechanisms, such as attenuated functions of GABAA and glycine receptors, decreased expression of enzymes for GABA synthesis or glycine transport, and a change of intracellular *versus* extracellular Cl^-^ gradients *via* downregulation of KCC2, as previously reviewed [6, 64, 67]. Other recently revealed disinhibition mechanisms include reduced inhibitory synapses onto the excitatory transmission neurons [40], and neuronal silencing *via* long-term potentiation of glycine receptor-mediated currents in GABAergic inhibitory neurons [68]. *In vivo* extracellular recordings have demonstrated that such disinhibition allows low-threshold inputs to activate normally nociception-specific neurons in both lamina I and lamina V, resulting in allodynia [64, 69–71]. The SOM lineage neurons are required to relay Aβ inputs from lamina III to lamina I under disinhibition conditions caused by the presence of bicuculline and strychnine to block GABA_A_ and glycine receptors [34], or following nerve injury (Cheng* et al.*, unpublished data). Consistently, mechanical allodynia induced by nerve injury or inflammation is virtually abolished following ablation of SOM lineage neurons in the dorsal horn [34]. Other studies have shown that inflammation and nerve injury open distinct allodynia pathways, based on differential c-Fos induction in Calb2^+^ neurons *versus* PKCγ^+^ neurons [41]. However, not all forms of neuronal activity or firing patterns lead to c-Fos induction. For example, low-threshold mechanical stimuli evoking touch perception under normal conditions rarely stimulate c-Fos in dorsal horn neurons [34]. As such, a lack of c-Fos induction in Calb2^+^ neurons following nerve lesions does not necessarily suggest that these neurons are not involved in the transmission of neuropathic pain, and a definite conclusion awaits *in vivo* or *ex vivo* recordings and/or imaging. It should also be pointed out that while activation of Calb2^+^ interneurons is sufficient to induce spontaneous nocifensive behaviors and mechanical allodynia [41], ablation of most of these neurons does not have a detectable impact on allodynia [34], possibly due to the existence of redundant allodynia pathways opened by inflammation and nerve injury.

The identities of A-LTMRs that transmit and/or gate mechanical pain are only beginning to be understood. Myelinated LTMRs expressing the toll-like receptor 5 [72] or delta opioid receptors [73, 74] are necessary for the expression of mechanical allodynia, and co-activation of LTMRs marked by MafA^Cre^ is able to attenuate acute pinprick pain mediated by Aδ mechanical nociceptors [54], possibly *via* feedforward activation of spinal inhibitory neurons for gate control. Not discussed in this review is the involvement in the induction and/or expression of mechanical allodynia of other types of primary afferents, such as C-LTMRs [75], MrgprD^+^ polymodal nociceptors [53], and sensitized fast-conducting myelinated mechanical nociceptors [76–78].

## Gate Control of Mechanical Itch

Itch, or pruritus, is defined as an unpleasant sensation associated with the desire to scratch [79]. The close connection between itch and scratching indicates that the neuronal apparatus for itch might initially have evolved as a nocifensive system to remove potentially harmful stimuli, such as insects moving across the skin (mechanical itch) or mosquitoes injecting pruritogens into the skin (chemical itch). This also highlights the inhibition of itch by painful stimuli, such as scratching, possibly *via* activation of spinal inhibitory neurons [80]. For example, pruritogen-evoked chemical itch is greatly sensitized (1) in mice with loss of a mixed population of spinal inhibitory interneurons whose development is dependent on the basic helix-loop-helix transcription factor Bhlhb5 [81, 82], and (2) in mice following ablation of glycinergic interneurons located deep in the dorsal horn [39]. Inhibition of chemical itch by painful stimuli is dependent on glutamate release from primary nociceptors, possibly *via* activation of the aforementioned inhibitory neurons [83, 84].

**Fig. 4:**
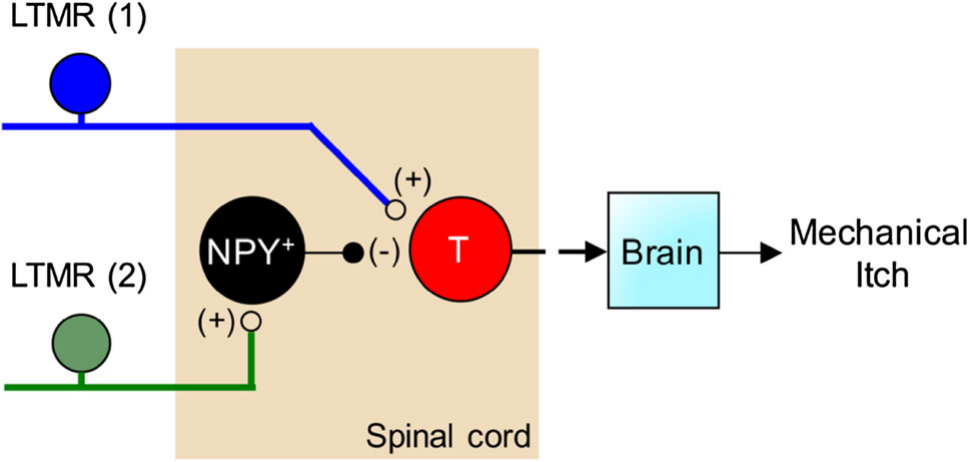
Schematic showing the gate control of mechanical itch. LTMR, low-threshold mechanoreceptor; T, a mechanical itch transmission neuron, which could be a projection neuron or an excitatory interneuron.

Regarding mechanical itch, the lightest stroking by a thin filament across the skin, particularly the upper lip [79], or vibration of a single facial venus hair, can evoke an intense itch sensation. Mechanical itch cannot be blocked by antagonists against histamine receptors [85]. However, a finger stroking with a slightly stronger force produces touch perception without itch. Thus, inputs from LTMRs not only gate mechanical pain, but also mechanical itch. Presumably, there is a class of itch-evoked LTMRs that are extremely sensitive to mechanical stimuli, but this low threshold mechanical itch can be gated (masked) *via* concurrent activation of other classes of LTMRs (Fig. 4). Recently, we found that spinal inhibitory neurons expressing neuropeptide Y::Cre (NPY::Cre) are required to gate touch-evoked mechanical itch [35]. NPY lineage neurons are enriched in laminae II–IV and most of them receive inputs from Aβ fibers. Ablation or silencing of the NPY::Cre-marked neurons within the dorsal spinal cord (and the dorsal hindbrain) causes the selective loss of gate control for mechanical itch, allowing low-threshold von Frey filament stimulation to evoke scratching responses, while pain and chemical itch sensitivity remains unchanged [35]. As a result, NPY::Cre neuron-ablated mice display excessive spontaneous scratching and eventual skin lesions [35]. Interestingly, this mechanical itch pathway is independent of GRPR^+^ spinal neurons that transmit chemical itch [29, 35, 86]. Furthermore, NPY neurons rarely overlap with Bhlhb5-dependent inhibitory neurons, which are required to gate chemical itch [81], suggesting distinct spinal microcircuits that transmit and gate mechanical *versus* chemical itch (Fig. 4).

## Concluding remarks

Extracellular recordings by Perl and his colleague first revealed many neurons in laminae I and II_o_ that are nociception-specific. Subsequent intracellular and genetic manipulations have now demonstrated that most of these neurons also receive monosynaptic and/or polysynaptic inputs from LTMRs, and LTMR inputs can either be silenced or become subthreshold due to feedforward activation of inhibitory neurons. Any given stimulus to the skin most likely activates a spectrum of sensory afferents with different receptive fields, conduction velocities, and adaptation rates, and the outcome of spinal transmission neurons in response to such a stimulus depends on the spatial and temporal summation of all of the excitatory and inhibitory inputs generated by these sensory afferents. Manifestation of allodynia or alloknesis can therefore be achieved *via* many different mechanisms, such as central sensitization of transmission neurons or reduced feedforward inhibition, or both.

Several outstanding issues remain to be addressed. First, while MrgprD^+^ and NPY2R^+^ nociceptors are critical for the transmission of light and intense mechanical information, respectively [53, 54], peripheral LTMRs and spinal excitatory neurons that transmit mechanical itch have not yet been characterized. The underlying mechanotransducers are also unclear. Piezo2, a rapidly-adapting, mechanically-activated ion channel transmits innocuous touch [87–91] and proprioception [87, 92], but is dispensable for the transmission of both acute mechanical pain in humans [87] and inflammation-induced mechanical allodynia in mice [88]. Second, it remains to be determined if the same or different subtypes of LTMRs provide inputs to spinal excitatory neurons for pain transmission *versus* inhibitory interneurons for gate control. Third, more effort should be directed to understanding how these gated spinal circuits are altered under distinct pathological conditions, since a loss of gate control appears to be the hallmark symptom seen in chronic pain or itch patients [23, 25]. Addressing these questions will eventually help to develop new therapeutic strategies to treat chronic pain and itch, such as by restoring the lost gate control.
